# The incidence of co-morbidities related to obesity and overweight: A systematic review and meta-analysis

**DOI:** 10.1186/1471-2458-9-88

**Published:** 2009-03-25

**Authors:** Daphne P Guh, Wei Zhang, Nick Bansback, Zubin Amarsi, C Laird Birmingham, Aslam H Anis

**Affiliations:** 1 Centre for Health Evaluation and Outcome Sciences, St Paul's Hospital, Vancouver, BC, Canada; 2 Department of Psychiatry, University of British Columbia, Vancouver, BC, Canada; 3 School of Population and Public Health, University of British Columbia, Vancouver, BC, Canada

## Abstract

**Background:**

Overweight and obese persons are at risk of a number of medical conditions which can lead to further morbidity and mortality. The primary objective of this study is to provide an estimate of the incidence of each co-morbidity related to obesity and overweight using a meta-analysis.

**Methods:**

A literature search for the twenty co-morbidities identified in a preliminary search was conducted in Medline and Embase (Jan 2007). Studies meeting the inclusion criteria (prospective cohort studies of sufficient size reporting risk estimate based on the incidence of disease) were extracted. Study-specific unadjusted relative risks (RRs) on the log scale comparing overweight with normal and obese with normal were weighted by the inverse of their corresponding variances to obtain a pooled RR with 95% confidence intervals (CI).

**Results:**

A total of 89 relevant studies were identified. The review found evidence for 18 co-morbidities which met the inclusion criteria. The meta-analysis determined statistically significant associations for overweight with the incidence of type II diabetes, all cancers except esophageal (female), pancreatic and prostate cancer, all cardiovascular diseases (except congestive heart failure), asthma, gallbladder disease, osteoarthritis and chronic back pain. We noted the strongest association between overweight defined by body mass index (BMI) and the incidence of type II diabetes in females (RR = 3.92 (95% CI: 3.10–4.97)). Statistically significant associations with obesity were found with the incidence of type II diabetes, all cancers except esophageal and prostate cancer, all cardiovascular diseases, asthma, gallbladder disease, osteoarthritis and chronic back pain. Obesity defined by BMI was also most strongly associated with the incidence of type II diabetes in females (12.41 (9.03–17.06)).

**Conclusion:**

Both overweight and obesity are associated with the incidence of multiple co-morbidities including type II diabetes, cancer and cardiovascular diseases. Maintenance of a healthy weight could be important in the prevention of the large disease burden in the future. Further studies are needed to explore the biological mechanisms that link overweight and obesity with these co-morbidities.

## Background

A substantial literature has emerged which has found that overweight and obesity are major causes of co-morbidities which can lead to further morbidity and mortality [1-3]. The related health care costs are substantial [4-6]. As the number of associated co-morbidities continues to increase, systematic reviews and meta-analysis are important tools to summarize the findings and produce more precise estimates of risk associated with overweight and obesity.

The primary objective of this study is to provide a comprehensive review of the incidence of co-morbidities related to obesity and overweight. We have identified a number of recent systematic reviews and meta-analyses on type II diabetes [7], cardiovascular diseases [8,9], cancer [10], breast cancer [11,12], esophageal or cardia adenocarcinoma [13], pancreatic cancer [14] and prostate cancer [15]. The rationale for re-conducting a review is threefold. Firstly, it has been reported that abdominal obesity, defined by waist circumference (WC) measurement in comparison to the more traditional obesity definition, based on Body Mass Index (BMI) measurement, is an even better predictor of many cardiovascular diseases and type II diabetes [16-24]. However, most recent reviews have only focused on obesity defined by BMI instead of WC. For example, a recent meta-analysis study has compared BMI and WC as risk factors for ischaemic heart disease and stroke but it only included studies with population from the Asia Pacific region [8]. Also, only meta-analysis studies on BMI and type II diabetes have been conducted [7].

Secondly, associating the incidence of co-morbidities with overweight and obesity can be done in many ways since there are many different definitions. For instance, many previous reviews have combined studies that have found the association with per unit change of BMI (kg/m^2^) and WC (cm) measurements [7-9,11,14,15]. We are interested in measuring the incidence by categorization of overweight and obesity defined by BMI and WC measurements, which has not been the focus of a majority of previous reviews.

Thirdly, the previous meta-analysis studies primarily focused on individual co-morbidities and they were conducted by different authors and using different search strategies, inclusion criteria and analysis methods. Only one recent meta-analysis study conducted by Katzmarzyk and Janssen comprehensively estimated the incidence of eight different chronic diseases associated with obesity [5]. An objective of this review is to apply a consistent methodology across all relevant co-morbidities. This enables us to compare the number of studies and size of effect across all co-morbidities.

## Methods

### Exposure variables

The definition for overweight is having a BMI greater than or equal to 25 kg/m^2 ^and below 30 kg/m^2^. The definition for obesity is having a BMI greater than or equal to 30 kg/m^2 ^[25,26]. According to the World Health Organization (WHO), the definition for abdominally overweight or obesity is a WC of greater than or equal to 80 cm and 88 cm, respectively, for females, and 94 cm and 102 cm, respectively, for males [25,26].

### Disease outcomes

Possible co-morbidities of overweight and obesity were identified from a preliminary search reviewed by an eating disorder and obesity expert and a review of previous systematic reviews [1-3]. We also reviewed the leading causes of global burden of disease and included the diseases reported with burden attributable to overweight and obesity [27]. Twenty co-morbidities were initially included in this analysis: cancer (kidney, colorectal, prostate, ovarian, uterine/endometrial, esophageal, pancreatic, and post-menopausal breast), type II diabetes, cardiovascular disease risk (hypertension, coronary artery disease, congestive heart failure, pulmonary embolism, stroke, dislipidaemia), gallbladder disease, chronic back pain, osteoarthritis, asthma, and sleep apnea.

### Literature review

A literature search was conducted using the search terms: 'Incidence, Prevalence, Risk, Risk Factors, Cohort Studies, Longitudinal Studies, Follow-up Studies, or Prospective Studies' in combination with 'Adipose Tissue, Obesity, Body Mass Index, or Body Composition' (all "exploded"). These same search terms were applied to each co-morbidity (also "exploded") for both Medline and Embase search engines to retrieve all potentially relevant English articles (until January, 2007). We also searched ISI Web of Science, Google Scholar, and the bibliographies of retrieved articles.

**Figure 1:**
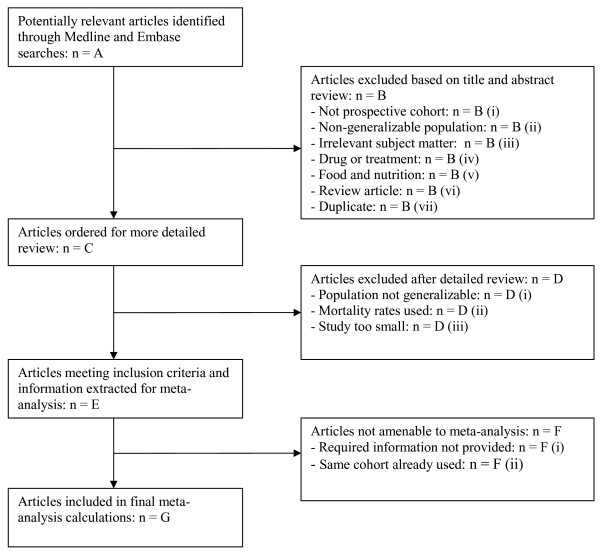
**Flowchart of article distribution for all diseases**.

The articles obtained from the literature search were then evaluated according to criteria set out in Figure 1. Criteria for inclusion were: prospective cohort study of the general population of a Western country (countries in Europe or North America, Australia or New Zealand), relevant outcomes, a sample size of at least 200 subjects, and risk estimate based on the incidence of disease instead of the mortality rate of disease. For large cohorts with multiple articles meeting the defined criteria, the most recent article or the article with the most usable information was used. Studies were excluded if they did not provide enough data to allow calculation of unadjusted relative risks (RRs) with 95% confidence intervals (CI) for the overweight and obese groups compared to the normal group. Data extracted for study characteristics included study design, country, cohort name, duration of follow-up, number of patients in each study group, age range, gender and ethnicity. Ascertainment of exposure and outcome variables was also recorded. The literature search was conducted by ZA, the decisions on inclusion and exclusion were made by ZA, NB, DPG, CLB and the data were extracted by ZA and DPG.

### Meta-analysis

RRs were measured by incidence rate ratios (IRRs) when person-time data were available and by the ratios of proportions (RR-Ps) when person-time data were not available. Study-specific unadjusted RRs on the log scale comparing overweight with normal and obese with normal were weighted by the inverse of their corresponding variances to obtain a pooled RR with 95% CIs. We used the random-effects model to estimate the pooled RR using the maximum likelihood estimation method [28]. The Q statistic was also calculated to assess the homogeneity of RRs (log scale) [28]. Potential publication bias was visually inspected by funnel plots and tested by asymmetry tests [29,30]; however it was performed only on those meta-analyses which included sufficient number of studies (N>5). We also did various sensitivity analyses stratified on the length of follow-up, age criteria and country to examine the robustness of the results.

WC measurements were considered to be the better risk predictor for some co-morbidities such as diabetes, hypertension, coronary artery disease, congestive heart failure, stroke and gallbladder disease [16-24]. Therefore, when the RRs based on WC measurements for these co-morbidities were available, they were pooled separately from those based on BMI and used as the final RRs for these co-morbidities. When both IRR and RR-P estimates were available, both estimates were presented. Final results of RRs were selected based on the number of pooled studies, the duration of study follow-up and the sample size of included studies

## Results

A total of 89 relevant and unique studies were identified; several studies featured for more than one co-morbidities. Of the 20 co-morbidities, 18 were identified to have at least one study meeting the inclusion criteria. Some studies were applicable to more than one co-morbidity. No studies were found for dislipidaemia and sleep apnea. The total numbers of studies included for each co-morbidity varied from 1 to 14. Reasons for exclusion are given in Table 1.

**Table 1:** Article distribution for all diseases (see Figure 1 for explanation)

**Diseases**	**A**	**B**	**B** **(i)**	**B** **(ii)**	**B** **(iii)**	**B** **(iv)**	**B** **(v)**	**B** **(vi)**	**B** **(vii)**	**C**	**D**	**D** **(i)**	**D** **(ii)**	**D** **(iii)**	**E**	**F**	**F** **(i)**	**F** **(ii)**	**G**
**Type II Diabetes**	8142	8075	6687	18	1216	20	28	18	88	67	37	17	6	14	30	21	19	2	9

**Colorectal cancer**	445	413	5	22	261	8	42	57	18	32	6	2	2	2	26	14	14	0	12

**Kidney cancer**	2661	2606	55	12	2010	51	50	127	301	55	43	16	18	9	12	7	7	0	5

**Prostate cancer**	491	462	28	1	258	0	37	52	86	29	14	5	9	0	15	7	6	1	8

**Breast cancer**	2755	2682	145	18	1793	19	110	195	402	73	43	6	34	3	30	16	13	3	14

**Ovarian cancer**	241	228	23	1	143	0	10	13	38	13	4	1	3	0	9	0	0	0	9

**Endometrial cancer**	1249	1192	103	17	729	8	30	105	200	57	41	27	3	11	16	6	5	1	10

**Pancreatic cancer**	155	131	5	0	81	0	14	7	24	24	15	5	10	0	9	3	2	1	6

**Esophageal cancer**	230	222	23	0	103	2	13	34	47	8	6	4	2	0	2	1	1	0	1

**Hypertension**	2882	2773	59	55	2256	53	78	81	191	109	88	23	53	12	21	17	17	0	4

**CAD**	3041	2966	96	17	2202	42	66	82	461	75	50	10	27	13	25	14	11	3	11

**CHF**	625	586	10	5	418	6	1	41	105	39	25	0	14	11	14	10	9	1	4

**PE**	527	497	48	13	321	1	0	24	90	30	11	3	3	5	19	18	18	0	1

**Stroke**	2783	2755	172	29	1747	17	24	234	532	28	17	6	5	6	11	4	4	0	7

**Asthma**	1408	1359	89	13	598	47	47	84	481	49	36	2	25	9	13	9	9	0	4

**GD**	319	305	29	9	175	0	7	32	53	14	5	0	2	3	9	5	5	0	4

**OA**	853	824	43	10	390	51	38	123	169	29	14	5	2	7	15	12	12	0	3

**CBP**	324	306	30	20	143	39	11	24	39	18	6	0	4	2	12	11	11	0	1

CAD: Coronary Artery Disease; CHF: Congestive Heart Failure; PE: Pulmonary Embolism; GD: Gallbladder Disease; OA: Osteoarthritis; CBP: Chronic Back Pain

The majority of the studies were conducted in US (55%) and in European countries (40%). Study characteristics such as age criteria, study follow-up, ascertainment of exposure and outcome variables were reported by the majority of the studies. However, only a small number of studies reported sample ethnicity and of those, the majority was US studies. Among those US studies, one study (endometrial cancer) was about the black women [31] while for the remaining US studies, the proportion of whites ranged from 81% to 95%. The mean duration of study follow-up was 12.5 (SD = 7.2) years. Over half of the studies (53%) were longer than 10 years while less than 10% of the studies were shorter than 5 years. BMI and WC measurements were clinically measured on 43% of the studies and were self-reported on 56% of the studies while one study did not provide such information. Regarding the ascertainment of cases, 43 (48%) studies identified cases from registry, database centre or clinical evaluation; 34 (38%) studies were based on subject self-reported information with some kind of confirmation method such as medical records review; 6 studies were based on medical records review and 5 studies (4 for asthma and 1 for type-2 diabetes) were relying on self-reported information alone. Note that cancer cases were identified from cancer registry/database on 66% of the studies.

Table 2 summarized our final results. Figures 2 to 18 presented the detailed results including study-specific and pooled estimates. Results from the meta-analysis were summarized in the following sections for each co-morbidity.

**Figure 2:**
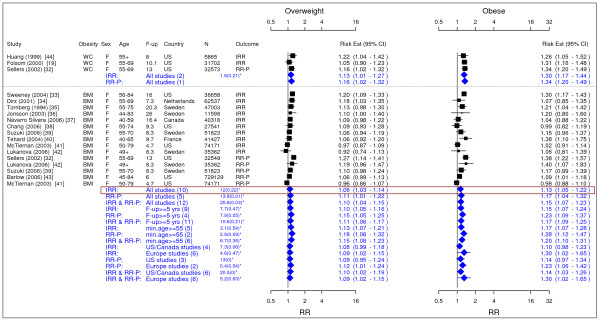
**Meta-analysis of studies for post menopausal breast cancer**. *Q-statistic(p-value); F-up is follow-up in years; square shape: study- and gender- specific risk estimates; diamond shape: pooled risk estimates.

**Figure 3:**
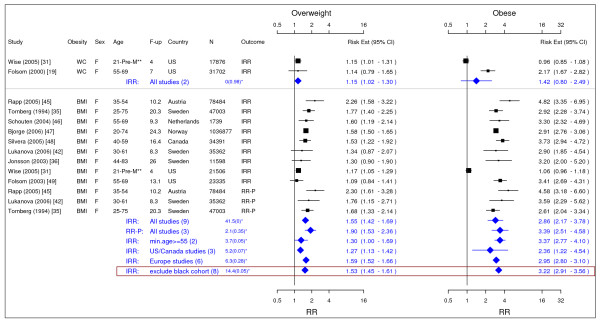
**Meta-analysis of studies for endometrial cancer**. *Q-statistic(p-value); **pre-menopause, square shape: study- and gender- specific risk estimates; diamond shape: pooled risk estimates.

**Figure 4:**
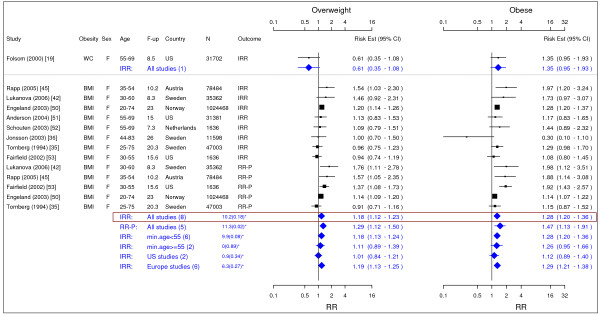
**Meta-analysis of studies for ovarian cancer**. *Q-statistic(p-value); F-up is follow-up in years; square shape: study- and gender- specific risk estimates; diamond shape: pooled risk estimates.

**Figure 5:**
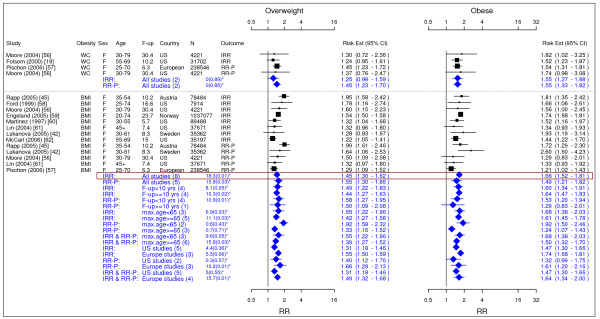
**Meta-analysis of studies for colorectal cancer-females**. *Q-statistic(p-value); F-up is follow-up in years; square shape: study- and gender- specific risk estimates; diamond shape: pooled risk estimates.

**Figure 6:**
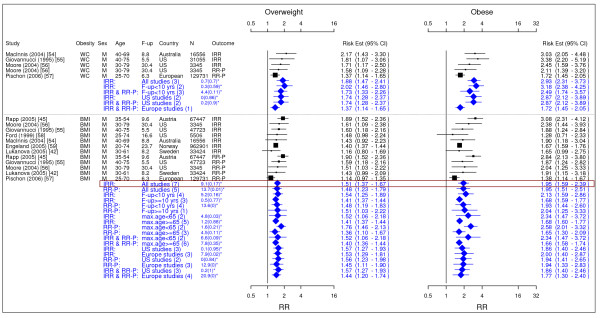
**Meta-analysis of studies for colorectal cancer-males**. *Q-statistic(p-value); F-up is follow-up in years; square shape: study- and gender- specific risk estimates; diamond shape: pooled risk estimates.

**Figure 7:**
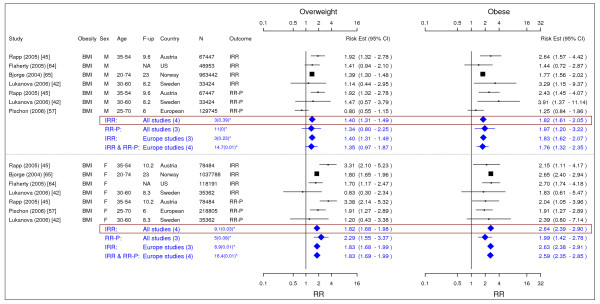
**Meta-analysis of studies for kidney cancer**. *Q-statistic(p-value); F-up is follow-up in years; square shape: study- and gender- specific risk estimates; diamond shape: pooled risk estimates.

**Figure 8:**
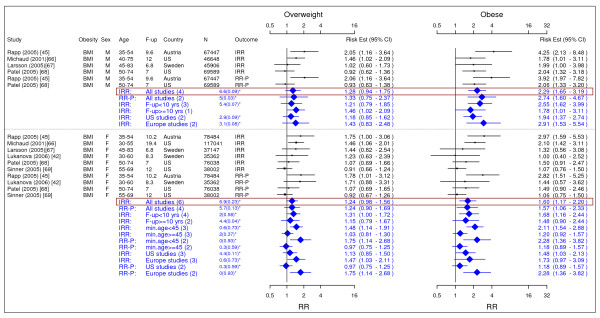
**Meta-analysis of studies for pancreatic cancer**. *Q-statistic(p-value); F-up is follow-up in years; square shape: study- and gender- specific risk estimates; diamond shape: pooled risk estimates.

**Figure 9:**
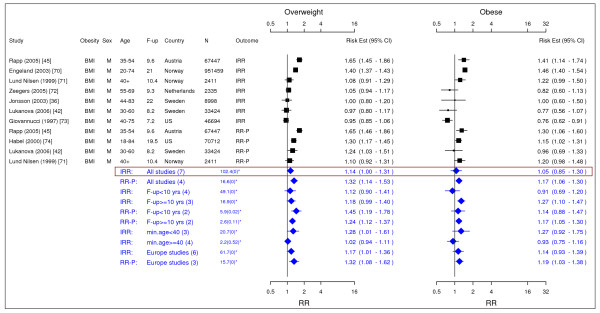
**Meta-analysis of studies for prostate cancer**. *Q-statistic(p-value); F-up is follow-up in years; square shape: study- and gender- specific risk estimates; diamond shape: pooled risk estimates.

**Figure 10:**
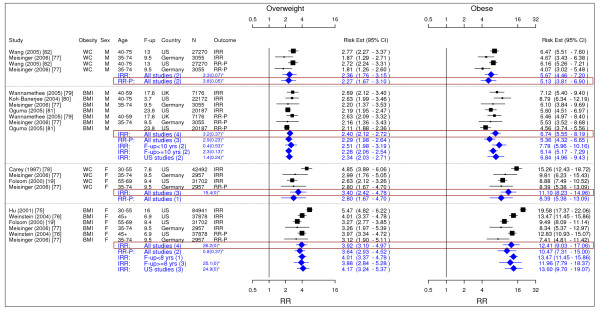
**Meta-analysis of studies for type II diabetes**. *Q-statistic(p-value); F-up is follow-up in years; square shape: study- and gender- specific risk estimates; diamond shape: pooled risk estimates.

**Figure 11:**
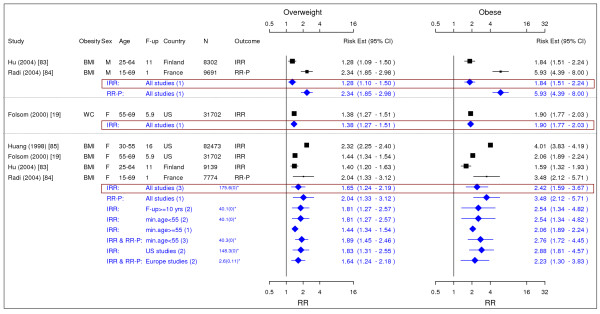
**Meta-analysis of studies for hypertension**. *Q-statistic(p-value); F-up is follow-up in years; square shape: study- and gender- specific risk estimates; diamond shape: pooled risk estimates.

**Figure 12:**
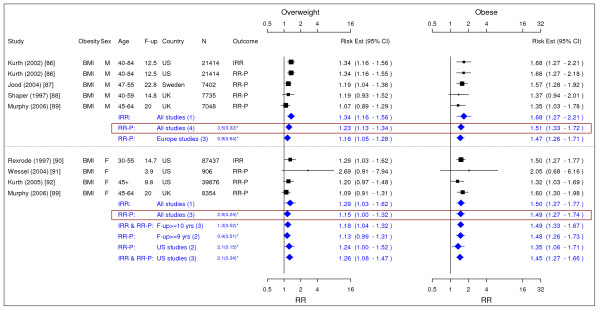
**Meta-analysis of studies for stroke**. *Q-statistic(p-value); F-up is follow-up in years; square shape: study- and gender- specific risk estimates; diamond shape: pooled risk estimates.

**Figure 13:**
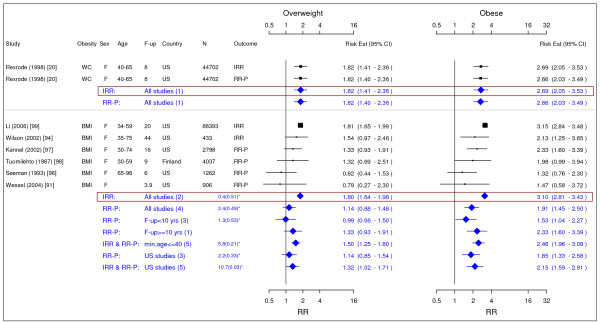
**Meta-analysis of studies for coronary artery disease-females**. *Q-statistic(p-value); F-up is follow-up in years; square shape: study- and gender- specific risk estimates; diamond shape: pooled risk estimates.

**Figure 14:**
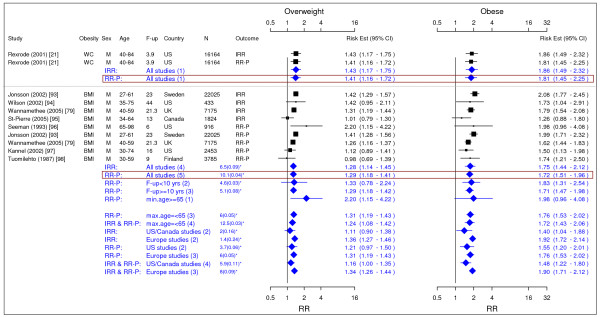
**Meta-analysis of studies for coronary artery disease-males**. *Q-statistic(p-value); F-up is follow-up in years; square shape: study- and gender- specific risk estimates; diamond shape: pooled risk estimates.

**Figure 15:**
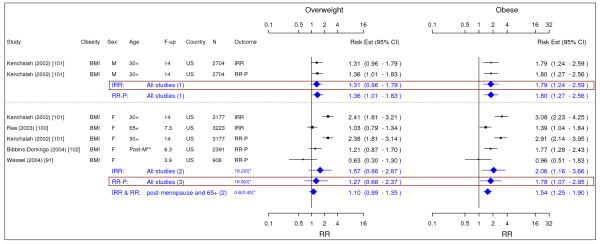
**Meta-analysis of studies for congestive heart failure**. *Q-statistic(p-value); **post-menopause; square shape: study- and gender- specific risk estimates; diamond shape: pooled risk estimates.

**Figure 16:**
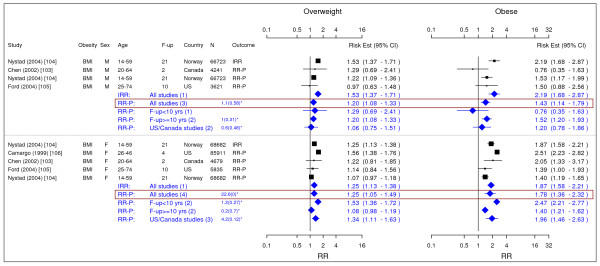
**Meta-analysis of studies for asthma**. *Q-statistic(p-value); F-up is follow-up in years; square shape: study- and gender- specific risk estimates; diamond shape: pooled risk estimates.

**Figure 17:**
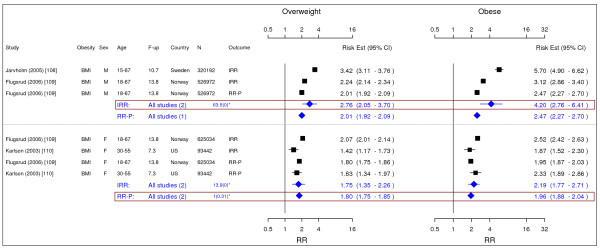
**Meta-analysis of studies for osteoarthritis**. *Q-statistic(p-value); F-up is follow-up in years; square shape: study- and gender- specific risk estimates; diamond shape: pooled risk estimates.

**Figure 18:**
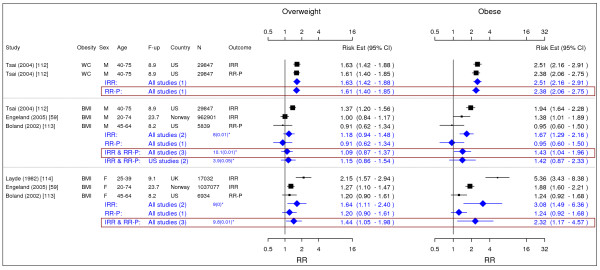
**Meta-analysis of studies for gallbladder disease**. *Q-statistic(p-value); F-up is follow-up in years; square shape: study- and gender- specific risk estimates; diamond shape: pooled risk estimates.

**Table 2:** Relative co-morbidity risks related to being overweight or obese

**Co-morbidity**	**Measure**	**Overweight**		**Obesity**	
		
		**Male**	**Female**	**Male**	**Female**
Type II Diabetes*	**BMI**	2.40 (2.12–2.72)	3.92 (3.10–4.97)	6.74 (5.55–8.19)	12.41 (9.03–17.06)

	**WC**	2.27 (1.67–3.10)^†^	3.40 (2.42–4.78)	5.13 (3.81–6.90)^†^	11.10 (8.23–14.96)

**Cancer**					

Breast, Postmenopausal	**BMI**	-	1.08 (1.03–1.14)	-	1.13 (1.05–1.22)

Colorectal	**BMI**	1.51 (1.37–1.67)	1.45 (1.30–1.62)	1.95 (1.59–2.39)	1.66 (1.52–1.81)

Endometrial	**BMI**	-	1.53 (1.45–1.61)	-	3.22 (2.91–3.56)

Esophageal	**BMI**	1.13 (1.02–1.26)	1.15 (0.97–1.36)	1.21 (0.97–1.52)	1.20 (0.95–1.53)

Kidney	**BMI**	1.40 (1.31–1.49)	1.82 (1.68–1.98)	1.82 (1.61–2.05)	2.64 (2.39–2.90)

Ovarian	**BMI**	-	1.18 (1.12–1.23)	-	1.28 (1.20–1.36)

Pancreatic	**BMI**	1.28 (0.94–1.75)	1.24 (0.98–1.56)	2.29 (1.65–3.19)	1.60 (1.17–2.20)

Prostate	**BMI**	1.14 (1.00–1.31)	-	1.05 (0.85–1.30)	-

**Cardiovascular Diseases**					

Hypertension*	**BMI**	1.28 (1.10–1.50)	1.65 (1.24–2.19)	1.84 (1.51–2.24)	2.42 (1.59–3.67)

	**WC**	NA	1.38 (1.27–1.51)	NA	1.90 (1.77–2.03)

Coronary Artery Disease*	**BMI**	1.29 (1.18–1.41)^†^	1.80 (1.64–1.98)	1.72 (1.51–1.96)^†^	3.10 (2.81–3.43)

	**WC**	1.41 (1.16–1.72)^†^	1.82 (1.41–2.36)	1.81 (1.45–2.25)^†^	2.69 (2.05–3.53)

Congestive Heart Failure*	**BMI**	1.31 (0.96–1.79)	1.27 (0.68–2.37)^†^	1.79 (1.24–2.59)	1.78 (1.07–2.95)^†^

Pulmonary Embolism	**BMI**	1.91 (1.39–2.64)	1.91 (1.39–2.64)	3.51 (2.61–4.73)	3.51 (2.61–4.73)

Stroke*	**BMI**	1.23 (1.13–1.34)^†^	1.15 (1.00–1.32)^†^	1.51 (1.33–1.72)^†^	1.49 (1.27–1.74)^†^

**Other**					

Asthma	**BMI**	1.20 (1.08–1.33)^†^	1.25 (1.05–1.49)^†^	1.43 (1.14–1.79)^†^	1.78 (1.36–2.32)^†^

Gallbladder Disease*	**BMI**	1.09 (0.87–1.37)^‡^	1.44 (1.05–1.98)^‡^	1.43 (1.04–1.96)^‡^	2.32 (1.17–4.57)^‡^

	**WC**	1.61 (1.40–1.85)^†^	NA	2.38 (2.06–2.75)^†^	NA

Osteoarthritis	**BMI**	2.76 (2.05–3.70)	1.80 (1.75–1.85)^†^	4.20 (2.76–6.41)	1.96 (1.88–2.04)^†^

Chronic Back Pain	**BMI**	1.59 (1.34–1.89)^†^	1.59 (1.34–1.89)^†^	2.81 (2.27–3.48)^†^	2.81 (2.27–3.48)^†^

BMI: body mass index; WC: waist circumference

^†^If indicated, the relative risks calculated from the ratios of proportions (RR-Ps) were used; otherwise, the incidence rate ratios (IRRs) were used;

^‡^Both RR-Ps and IRRs were used

*WC measures were considered to be the better risk predictor than BMI measures

Cancer: cases, not mortality and indicated by physician diagnosis of cancer; Coronary Artery Disease: indicated by Myocardial Infarction or Angina; Osteoarthritis: indicated by joint replacement; Chronic Back Pain: indicated by early retirement due to back pain

NA: Not available; "-" Not applicable

### Breast cancer

In total, 14 studies were identified for post menopausal breast cancer (Figure 2) [19,32-44]. The pooled IRRs [95% CI] across categories of WC were 1.13 [1.01–1.07] for overweight and 1.30 [1.17–1.44] for obesity while across categories of BMI the IRRs were 1.08 [1.03–1.14] for overweight and 1.13 [1.05–1.22] for obesity.

### Endometrial cancer

In total, 10 studies were identified to meet the inclusion criteria for endometrial cancer (Figure 3) [19,31,35,36,42,45-49]. The study on the US black women was not included in the final result as it showed systematic difference from other studies mainly on Caucasian population [31]. The pooled IRRs [95% CI] across categories of WC were 1.15 [1.02–1.30] for overweight and 1.42 [0.80–2.49] for obesity while across categories of BMI IRRs were 1.53 [1.45–1.61] for overweight and 3.22 [2.91–3.56] for obesity.

### Ovarian cancer

In total, 9 studies were identified for ovarian cancer (Figure 4) [19,35,36,42,45,50-53]. The single IRR [95% CI] across categories of WC were 0.61 [0.35–1.08] for overweight and 1.35 [0.95–1.93] for obesity while pooled IRRs estimates across categories of BMI were 1.18 [1.12–1.23] for overweight and 1.28 [1.20–1.36] for obesity.

### Colorectal cancer

A total of 12 studies were identified for colorectal cancer (Figures 5 and 6) [19,42,45,54-62]. For men, the pooled IRRs [95% CI] across categories of WC were 1.88 [1.47–2.41] for overweight and 2.93 [2.31–3.73] for obesity and across categories of BMI were 1.51 [1.37–1.67] for overweight and 1.95 [1.59–2.39] for obesity. For women, the pooled IRRs across categories of WC were 1.25 [0.98–1.59] for overweight and 1.55 [1.27–1.88] for obesity and those across categories of BMI were 1.45 [1.30–1.62] for overweight and 1.66 [1.52–1.81] for obesity.

### Esophageal cancer

Only 1 study was identified for esophageal cancer [63]. The study found the risk of cancer based on overweight to be 1.15 [0.97–1.36] and 1.13 [1.02–1.26] for females and males, respectively. The corresponding risks for obesity were 1.20 [0.95–1.53] and 1.21 [0.97–1.52].

### Kidney cancer

We identified 5 studies meeting the inclusion criteria relating overweight and obesity to kidney cancer (Figure 7) [42,45,57,64,65]. The pooled IRRs [95% CI] across categories of BMI for men were 1.40 [1.31–1.49] for overweight and 1.82 [1.61–2.05] for obesity. For women the corresponding risks were 1.82 [1.68–1.98] and 2.64 [2.39–2.90].

### Pancreatic cancer

The search identified 6 studies giving information on the risk of pancreatic cancer attributable to overweight and obesity (Figure 8) [42,45,66-69]. The pooled IRRs [95% CI] across categories of BMI for men were 1.28 [0.94–1.75] for overweight and 2.29 [1.65–3.19] for obesity. For women the corresponding risks were 1.24 [0.98–1.56] and 1.60 [1.17–2.20].

### Prostate cancer

The search identified 8 studies giving information on the risk of prostate cancer attributable to overweight and obesity (Figure 9) [36,42,45,70-74]. The pooled IRRs [95% CI] across categories of BMI were 1.14 [1.00–1.31] for overweight and 1.05 [0.85–1.30] for obesity.

### Type II diabetes

Nine studies met the inclusion criteria and were included in the meta-analysis (Figure 10) [19,75-82]. In general, elevated BMI and WC were significantly associated with type II diabetes in men and women. The pooled IRRs [95% CI] across categories of BMI were 2.40 [2.12–2.72] and 6.74 [5.55–8.19] in men while the corresponding IRRs in women were 3.92 [3.10–4.97] and 12.41 [9.03–17.06]. The association between increased WC and type II diabetes was similar but weaker in comparison with BMI. Only two studies were included in men. The pooled IRRs [95% CI] across categories of WC were 2.36 [1.76–3.15] and 5.67 [4.46–7.20] in men and the pooled RR-Ps [95% CI] based on the same two studies were 2.27 [1.67–3.10] and 5.13 [3.81–6.90], respectively. The pooled RR-Ps were more conservative RR estimates and presented in the summary table (Table 2). The pooled IRRs [95% CI] across categories of WC were 3.40 [2.42–4.78] and 11.10 [8.23–14.96] in women.

### Hypertension

Four studies met the inclusion criteria and were included in the meta-analysis (Figure 11) [19,83-85]. The pooled IRR [95% CI] estimates for hypertension across categories BMI for men were 1.28 [1.10–1.50] for overweight and 1.84 [1.51–2.24] for obesity. The corresponding figures for females were 1.65 [1.24–2.19] and 2.42 [1.59–3.67]. The single IRR estimate based on WC for women was 1.38 [1.27–1.51] for overweight and 1.90 [1.77–2.03] for obesity.

### Stroke

Seven studies met the inclusion criteria and were included in the meta-analysis (Figure 12) [86-92]. The pooled RR-P [95% CI] estimates for stroke across categories BMI for men were 1.23 [1.13–1.34] for overweight and 1.51 [1.33–1.72] for obesity. The corresponding results for females were 1.15 [1.00–1.32] and 1.49 [1.27–1.74].

### Coronary Artery Disease

Eleven studies were identified with evidence for coronary artery disease related to obesity (Figures 13 and 14) [20,21,79,91,93-99]. The pooled RR-P estimates for coronary artery disease across categories of WC were more conservative RR estimates for men than the corresponding IRR estimates and thus were presented in the summary table (Table 2). The RR-P [95% CI] estimates for WC were 1.41 [1.16–1.72] for overweight and 1.81 [1.45–2.25] for obesity. The corresponding results for BMI were 1.29 [1.18–1.41] and 1.72 [1.51–1.96]. While the pooled RR-P estimates based on BMI measurements for women were generated from 4 studies, the IRR estimates were generated from 2 different studies with longer follow-up. Thus, the IRR estimates were the RR estimates for women presented in the summary table (Table 2). The estimates were 1.80 [1.64–1.98] for overweight and 3.10 [2.81–3.43] for obesity based on BMI measurements and 1.82 [1.41–2.36] and 2.69 [2.05–3.53] for WC.

### Congestive Heart Failure

Four studies were identified with evidence for congestive heart failure related to obesity (Figure 15) [91,100-102]. The pooled IRR [95% CI] estimates for congestive heart failure across categories of BMI for men were 1.31 [0.96–1.79] for overweight and 1.79 [1.24–2.59] for obesity. The pooled RR-P estimates for females based on 3 studies were 1.27 [0.68–2.37] and 1.78 [1.07–2.95], which were chosen as the RR estimates over the IRR estimates based on 2 studies.

### Asthma

Four studies were identified with evidence for asthma related to obesity (Figure 16) [103-106]. The pooled RR-P [95% CI] estimates for asthma across categories of BMI for men were 1.20 [1.08–1.33] for overweight and 1.43 [1.14–1.79] for obese. The corresponding numbers for females were 1.25 [1.05–1.49] and 1.78 [1.36–2.32].

### Chronic back pain

Only 1 study was identified to meet the inclusion criteria for chronic back pain [107]. The study identified the association for the overweight and obesity with early retirement due to chronic back pain. The study found RR-P [95% CI] estimates across categories of BMI of chronic back pain based on overweight to be 1.59 [1.34–1.89] and for obesity 2.81 [2.27–3.48].

### Osteoarthritis

We identified three studies meeting the inclusion criteria relating overweight and obesity to osteoarthritis (Figure 17) [108-110]. The studies identified the risk of joint replacement attributable to being overweight and obese. The pooled IRRs [95% CI] across categories of BMI for men were 2.76 [2.05–3.70] for overweight and 4.20 [2.76–6.41] for obesity. For women, the RR-P estimates were more conservative RR estimate and they were 1.80 [1.75–1.85] and 1.96 [1.88–2.04].

### Pulmonary embolism

Only 1 study was identified to meet the inclusion criteria for pulmonary embolism [111]. The study found IRR [95% CI] across categories of BMI of Pulmonary embolism based on overweight to be 1.91 [1.39–2.64] and for obesity 3.51 [2.61–4.73].

### Gallbladder disease

We identified four studies meeting the inclusion criteria relating overweight and obesity gallbladder disease (Figure 18) [59,112-114]. The pooled IRRs [95% CI] across categories of WC for men were 1.63 [1.42–1.88] for overweight and 2.51 [2.16–2.91] for obesity. The corresponding pooled RR-Ps were 1.61 [1.40–1.85] and 2.38 [2.06–2.75]. Thus, the pooled RR-Ps were more conservative RR estimates and presented in Table 2. Across categories of BMI, the pooled IRR and RR-P estimates were presented as the RR estimates in Table 2 and they were 1.09 [0.87–1.37] for overweight and 1.43 [1.04–1.96] for obesity. For women only estimates for BMI were identified giving pooled IRR and RR-P estimates of 1.44 [1.05–1.98] for overweight and 2.32 [1.17–4.57] for obesity.

Potential publication bias was assessed for post-menopausal breast cancer, endometrial cancer, ovarian cancer, colorectal cancer, pancreatic cancer for females and prostate cancer. We found some evidence of funnel-plot asymmetry for obesity in prostate cancer where bigger studies tended to show stronger positive association than smaller studies. No evidence of publication bias was found in the other meta-analyses.

Our sensitivity analyses showed that our results were in general robust with the following exceptions. For ovarian cancer, associations for both overweight and obesity were slightly weaker in US studies compared to European studies. Similar country differences were found in pancreatic cancers; in addition, weaker associations were observed in older population. Studies with shorter follow-up time and of older population showed slightly weaker association of obesity with prostate cancer. In coronary artery disease for females, studies with shorter follow-up showed weaker associations of both overweight and obese. In coronary artery disease for males, weaker associations were observed in US and Canadian studies. Studies of post-menopausal and senior women on congestive heart failure showed weaker associations for both overweight and obesity.

## Discussion

We have comprehensively reviewed 20 co-morbidities for high quality cohort studies which determine risk factors associated with overweight or obesity. 18 co-morbidities were identified and meta-analysis was performed where at least 1 study was found. A summary of the results can be found in Table 2.

There are a number of alternative meta-analyses with which we can compare our results. For example, recent meta-analyses have been reported in diabetes [115,116], cardiovascular diseases [117], coronary heart disease [118], hypertension [116], cancer [119], colorectal cancer [120-122], gallbladder cancer [123], pancreatic cancer [124], ovarian cancer [125] and asthma [126]. However, each study uses different definitions of overweight and obesity, includes varying quality of study designs, uses different methods for meta-analysis and ultimately only focuses on individual co-morbidities. Hence, the objective of our study is not only to provide up to date estimates of the risk of all possible co-morbidities attributable to overweight and obesity, but also to do it using consistent definitions and methodology.

In assessing whether obesity is related to a given co-morbidity, the occurrence timing of co-morbidities with respect to exposure of obesity is important in determining the causal pathway. Therefore, we included only the prospective cohort studies and excluded the cross-sectional studies and case control studies to minimize the associated potential biases. In addition, WC measurements were considered to be the better risk predictor for type II diabetes, hypertension, coronary artery disease, congestive heart failure, stroke and gallbladder disease [16-24]. In our studies, the risk for type II diabetes, female hypertension, coronary artery disease, and male gallbladder disease were estimated based on WC measurements.

Some limitations are worthy of further consideration. Firstly, other variables not included in our analysis might potentially confound our results. Most important is the exclusion of the level of physical inactivity which is a known risk factor for some co-morbidities and related to overweight and obesity [127]. Physical inactivity is often poorly reported and requiring its inclusion would have reduced the number of included studies. Secondly, for certain co-morbidities, we only identified 1 or 2 prospective cohort studies that adopted the WC measurements as the risk predictor. Further studies are required to determine the association between WC and some co-morbidities before an estimate of the risk can be calculated through a meta-analysis. Thirdly, given the sizable literature and that we were searching for non RCT studies for which search filters are more complex, we determined to use Medline and Embase as the electronic databases, and complement the search with checking reference lists and thorough searching the internet. We did not search other databases such as CINHAL, HealthSTAR, AMED, and BIOSIS. Therefore, bias might have occurred due to our search strategy. However, given the nature of the studies we are looking for, i.e., prospective cohort studies with high quality, we consider our search within Medline and Embase sufficient. Lastly, due to the small number of studies for most co-morbidities, assessment of potential publication bias was infeasible. However, we did not find evidence of publication bias in those meta-analyses where the number of studies was relatively large except for prostate cancer.

## Conclusion

In conclusion, this study provides a comprehensive estimate of the incidence of 18 co-morbidities attributable to overweight and obesity using standardized and consistent definitions and methodologies. Our findings confirm that overweight and obesity carry a profound health burden and will have a significant impact on health expenditures.

## Abbreviations

WC: Waist Circumference; BMI: Body Mass Index; RR: Relative Risk; IRR: Incidence Rate Ratio; RR-P: Relative Risk calculated from the ratio of Proportions; CI: Confidence Interval; WHO: World Health Organization.

## Competing interests

This manuscript is part of larger project funded by sanofi-aventis Canada Inc.

## Authors' contributions

ZA conducted literature search. ZA, NB, DPG, CLB evaluated the study articles and made decisions on inclusion and exclusion of the articles. DPG, WZ, AHA performed statistical analyses. All authors (DPG, WZ, NB, ZA, CLB, AHA) were involved in the manuscript development and its revision. All authors read and approved the final manuscript.

## Pre-publication history

The pre-publication history for this paper can be accessed here:


